# *Mycobacterium conceptionense* Pneumonitis in Patient with HIV/AIDS^1^

**DOI:** 10.3201/eid2510.190444

**Published:** 2019-10

**Authors:** Sarah M. Michienzi, Rodrigo M. Burgos, Richard M. Novak

**Affiliations:** University of Illinois at Chicago College of Pharmacy, Chicago, Illinois, USA (S.M. Michienzi, R.M. Burgos);; University of Illinois at Chicago College of Medicine, Chicago (R.M. Novak)

**Keywords:** Mycobacterium conceptionense, bacteria, nontuberculous mycobacteria, tuberculosis and other mycobacteria, pneumonitis, pneumonia, immunocompromised host, HIV/AIDS and other retroviruses

## Abstract

Approximately 21 human cases of infection with *Mycobacterium conceptionense* have been reported. However, most cases were outside the United States, and optimal treatment remains uncertain. We report a case of *M. conceptionense* pneumonitis in a patient with HIV/AIDS in the United States. The patient was cured with azithromycin and doxycycline.

*Mycobacterium conceptionense* is a nonpigmented, rapidly growing, nontuberculous mycobacterium, first isolated in France in 2006 (*1*). Approximately 21 cases of human infection have been reported (*1*–*10*). However, excluding the case we report here, only 2 cases have been reported in the United States (*2*). Optimal treatment for *M. conceptionense* infection remains uncertain. We report the clinical course and management of *M. conceptionense* pneumonitis in a patient with HIV/AIDS in the United States.

A 47-year-old black cisgender man sought care at an emergency department during 2015 for cough, shortness of breath, and diarrhea. He denied travel outside of the United States. The patient had HIV/AIDS, which was diagnosed during the 1980s but was untreated until this admission. He also had chronic hepatitis C, which was diagnosed during this admission. He was positive for HLA-B*5701, indicating hypersensitivity to the antiretroviral drug abacavir, but had no other known allergies to medications.

At admission, the patient was febrile (temperature 38.9°C) and had tachycardia (heart rate 112 beats/min) with low oxygen saturation (92% on room air), bibasilar rales, and poor inspiratory effort. Baseline laboratory test values were compiled (Table). A baseline chest radiograph showed increased interstitial marking and bibasilar patchy opacities. A baseline chest computed tomography scan showed bilateral interstitial and ground-glass opacities and a 6-mm nodule in the right middle lobe.

**Table:** Pertinent baseline laboratory test results for a 47-year-old man with *Mycobacterium conceptionense* pneumonitis and HIV/AIDS, United States*

Laboratory test	Value or result
Serum creatinine	0.89 mg/dL
Aspartate aminotransferase	25 U/L
Alanine aminotransferase	23 U/L
HIV RNA	**25,611 copies/mL**
CD4 cells	**19 cells/uL (5%)**
Hepatitis C virus antibody	Positive
Leukocytes	**2.3 × 10^3^/μL**
Neutrophils	1.9 × 10^3^/μL
Lymphocytes	**0.2 × 10^3^/μL**
Lactate dehydrogenase	**546 U/L**
*Histoplasma* antigen	Negative
Rapid plasma reagin	1:0
*Clostridioides difficile*	Negative
Stool culture	Negative

*Abnormal values are indicated in bold.

The patient was given empiric antimicrobial drugs (azithromycin 250 mg/d and ceftriaxone 1 g/d, both intravenously [IV]) for presumptive community-acquired pneumonia and trimethoprim/sulfamethoxazole (TMP/SMX; 800/160 mg every 6 h IV) for presumptive *Pneumocystis jirovecii* pneumonia (PJP). On day 4, ceftriaxone and azithromycin were discontinued. Induced sputum culture obtained on day 2 showed acid-fast bacilli (AFB) on day 8.

Infection with *M. tuberculosis* was not suspected because of the patient’s clinical manifestations and fast growth of the organism. The symptoms improved after admission. On day 11, he was discharged from the hospital and received oral TMP/SMX equivalent to that for intravenous dosing for PJP treatment. In addition, he erroneously received oral azithromycin (1,250 mg/wk) for *M. avium* complex prophylaxis.

On day 22, the patient returned to the ambulatory care clinic at the same institution. At this time, additional induced sputum cultures from days 3 and 4 were positive for AFB. His TMP/SMX treatment course was completed and decreased to 800/160 mg/day orally for secondary PJP prophylaxis. Azithromycin was corrected to treatment doses and increased to 250 mg/d orally. Baseline HIV genotyping showed wild-type virus, and antiretroviral therapy (ART) was initiated with elvitegravir/cobicistat/emtricitabine/tenofovir alafenamide (E/c/F/TAF) in a fixed-dose combination.

At day 43, the pneumonitis had clinically resolved, and repeat computed tomography and AFB culture showed negative results. A diagnosis of infection with *M. conceptionense* was confirmed from 3 induced sputum cultures obtained during days 2–4. Growth of *M. conceptionense* was identified by *rpoB* gene sequencing. Testing was performed at National Jewish Mycobacteriology Reference Laboratory (Denver, CO, USA). Drug susceptibility testing was not performed. An environmental source of the infection was not sought. Doxycycline (100 mg 2×/day orally) was given in addition to azithromycin because of lack of susceptibility information and previous case reports using dual therapy, although there is no clear guidance for management. ART with E/c/F/TAF was continued.

The patient is still profoundly immunosuppressed (CD4 cell count 60 cells/μL [6%]) because of nonadherence to ART. Darunavir (800 mg/day orally) was added to E/c/F/TAF because of development of resistance to ART, most notably the M184V pathway. We plan to continue oral azithromycin and doxycycline at current doses until immune reconstitution is achieved.

Cases of infection with *M. conceptionense* have been reported in immunocompetent and immunocompromised patients and in traumatic (e.g., after surgery or injury) and nontraumatic situations (*1*–*10*). The lungs are the most common site for *M. conceptionense* infection, comprising 7 of the ≈21 cases reported (*1*–*4*). Our patient was immunocompromised because of infection with HIV. Pathogen entry occurred by inhalation in a nontraumatic fashion and led to pneumonitis.

Outside the United States, *M. conceptionense* infection has been reported in France, Iran, Taiwan, South Korea, China, and Japan (*1*,*3*–*10*). The only 2 previously reported case-patients with *M. conceptionense* infection in the United States were also in Chicago but were epidemiologically unrelated to the patient we describe (*2*).

Similar to other reported case-patients, this patient was given broad-spectrum antimicrobial drugs, which were tailored once diagnosis of nontuberculous mycobacterium was confirmed. In vitro drug susceptibility data from rapidly growing mycobacteria indicate that *M. conceptionense* is susceptible to clarithromycin, doxycycline, and fluoroquinolones but resistant to sulfamethoxazole (*3*). In addition, macrolides, fluoroquinolones, or doxycycline have been used for treatment of *M. conceptionense* infections in case reports (*1*–*10*). These cases have assisted our choice of treatment for this case. In summary, our case report shows clinical and microbiological cure of *M. conceptionense* pneumonitis by using azithromycin and doxycycline in a patient with HIV/AIDS in the United States.
